# Current and emerging medications for the management of obesity in adults

**DOI:** 10.5694/mja2.51871

**Published:** 2023-03-19

**Authors:** Rosalind Walmsley, Priya Sumithran

**Affiliations:** ^1^ University of Melbourne Melbourne VIC; ^2^ Austin Health Melbourne VIC

**Keywords:** Obesity, Pharmaceuticals

**Summary**

- Obesity affects nearly one‐third of adults in Australia and is the second leading contributor to the nation's burden of disease.
- For people with obesity, weight loss of 5% or more has health benefits, and greater weight loss is associated with progressive improvements in health and health‐related quality of life.
- Long term management is required to sustain the health benefits of weight loss.
- In Australia, medications for obesity management are indicated in conjunction with lifestyle interventions in adults with obesity (body mass index ≥ 30 kg/m^2^) or those who are overweight (body mass index ≥ 27 kg/m^2^) with at least one complication of excess weight.
- Five medications are currently approved by the Therapeutic Goods Administration for obesity management in Australia, and the treatment pipeline is evolving rapidly, with several new agents under development for the management of obesity and its complications.

The prevalence of obesity among adults has nearly tripled worldwide since 1975 to more than 650 million in 2016,^1^ including 31% of adults in Australia.^2^ Obesity is the second leading contributor to Australia's burden of disease due to its complications, which include cardiovascular disease, type 2 diabetes, chronic kidney disease, fatty liver, obstructive sleep apnoea, and several cancers.^2^ Many of these complications can be prevented or mitigated with a loss of as little as 5% of total body weight (Box 1), with greater weight loss usually resulting in progressive improvements in obesity‐related complications and quality of life.^4^, ^5^, ^6^

Box 1 **Magnitude of weight loss required for improvement in obesity complications^3^**

**Figure:**
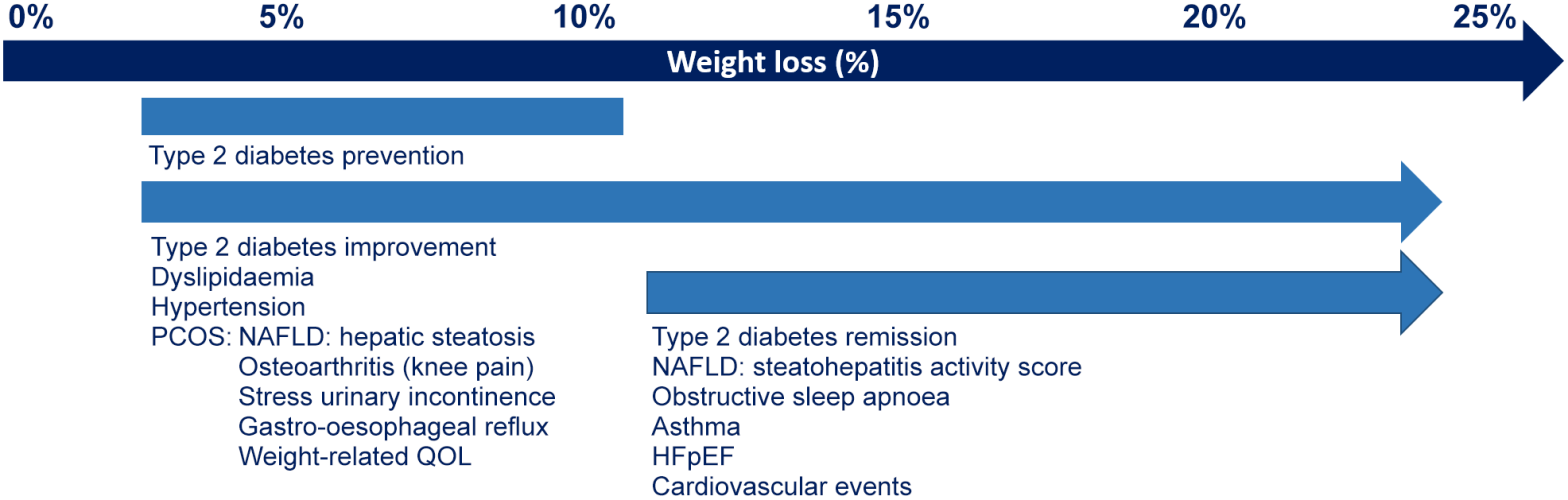
HFpEF = heart failure with preserved ejection fraction; NAFLD = non‐alcoholic fatty liver disease; PCOS = polycystic ovary syndrome; QOL = quality of life.

Lifestyle interventions that incorporate reduction in energy intake, improved diet quality and increased physical activity are the foundation of obesity management. However, obesity is chronic and relapsing, and most people who lose weight with lifestyle interventions alone will regain weight over time.^7^ Long term maintenance of weight loss is very challenging for most people because of complex interactions between biology, behaviour and the obesogenic environment. Weight loss leads to numerous long‐lasting biological changes, including a reduction in total energy expenditure greater than expected for the amount of lean mass lost, an increase in appetite, alterations in several hormones (including adipocyte, thyroid and gut hormones) involved in hunger, satiety and energy expenditure, and alterations in neural activity in several brain areas that mediate food intake.^8^, ^9^ Although direct links with weight regain have not been shown, many of these changes would appear to favour regain of lost weight. Lack of understanding of the biology of obesity perpetuates the misconception that it is simply due to lifestyle factors and inadequate motivation for behaviour change. This stigma is common, even in the health care sector, and can lead to reluctance by people with obesity to seek treatment, as well as compromise the quality of care provided (eg, incomplete discussion of treatment options).^10^

Clinical practice guidelines for obesity management recommend consideration of very low energy diets, medications and bariatric surgery when lifestyle interventions alone have not achieved therapeutic goals.^11^, ^12^ Growing recognition of the pathophysiology of obesity^12^, ^13^ has led to considerable advances in obesity pharmacotherapies over the past decade. This narrative review provides an overview of current and emerging medications for obesity management, including recommendations and knowledge gaps regarding their use in clinical practice.

## Methods

We searched the MEDLINE (Ovid) online database and the ClinicalTrials.gov registration to identify relevant studies, using the search terms “obesity”, “morbid obesity”, “overweight”, “body weight”, “weight loss”, “weight gain”, “adiposity”, “adipose”, “medication”, “pharmacotherapy” and “weight management”. We considered for inclusion human studies published in English before 22 August 2022 based on relevance, originality and impact (eg, number of citations), and screened the reference lists of relevant articles. Publications reporting outcomes of phase 3 clinical trials of obesity medications were preferentially included, along with any article otherwise known to the authors relevant to the topic and not identified through the search or reference list screen.

## Medications for obesity management

In Australia, medications, in conjunction with lifestyle interventions, are indicated for weight management in adults (≥18 years of age) with obesity (body mass index ≥ 30 kg/m^2^) or those who are overweight (body mass index ≥ 27 kg/m^2^, or ≥ 25 kg/m^2^ for phentermine) with at least one weight‐related complication. Five agents are currently approved by the Therapeutic Goods Administration (TGA) for obesity management: orlistat, phentermine, naltrexone–bupropion, and the glucagon‐like peptide 1 (GLP‐1) receptor agonists liraglutide and semaglutide. Their mechanisms of action, dosing, effects, and costs are summarised in Box 2. All currently approved medications, apart from orlistat, act centrally in brain regions involved in appetite regulation to increase satiety, reduce hunger, and, in some cases, reduce the rewarding properties of high calorie food. At present, the Pharmaceutical Benefits Scheme does not subsidise medications indicated for obesity management. In Australia, at the time of writing, semaglutide 2.4 mg weekly is indicated for chronic weight management but is not yet available.

Box 2 **Medications indicated for the treatment of obesity in adults in Australia**

**Table:** 

Medication	Phentermine*	Orlistat^†^	Liraglutide 3 mg^‡^	Naltrexone–bupropion^§^	Semaglutide 2.4 mg^¶^
Year of TGA approval	1991	2000	2015	2018	2022
Route and form	Oral (capsule)	Oral (tablet)	Subcutaneous (injection)	Oral (tablet)	Subcutaneous (injection)
Recommended dose	15 mg, 30 mg or 40 mg once daily	120 mg three times a day, with meals	Starting dose 0.6 mg daily, escalating by 0.6 mg per week over five weeks to 3 mg once daily	Starting dose one 8 mg naltrexone–90 mg bupropion tablet daily, escalating by one tablet per week over four weeks to two tablets twice daily (16 mg naltrexone–180 mg bupropion twice a day)	Starting dose 0.25 mg weekly, escalating every four weeks to 2.4 mg weekly over 16 weeks
Mechanism of action for weight loss	Reduces appetite by stimulating neural release of noradrenaline, serotonin and dopamine	Reduces absorption of dietary fat by inhibiting gastric and pancreatic lipases	Reduces appetite by stimulating GLP‐1 receptors in several brain areas	Reduces appetite by stimulating activity of POMC neurons in the hypothalamus	Reduces appetite by stimulating GLP‐1 receptors in several brain areas
Contraindications and precautions	Coronary artery disease, uncontrolled hypertension, hyperthyroidism, glaucoma, cardiac arrhythmias, MAOI, pregnancy, breastfeeding	Pregnancy, breastfeeding	Personal or family history of medullary thyroid carcinoma or multiple endocrine neoplasia syndrome type 2, history of pancreatitis, pregnancy, breastfeeding	Uncontrolled hypertension, seizure disorders, bipolar disorder, undergoing abrupt discontinuation of alcohol or anticonvulsant drugs, chronic opioid use, MAOI, pregnancy, breastfeeding	As for liraglutide
	Not recommended with SSRIs				
Adverse effects	Dry mouth, insomnia, palpitations, tachycardia, hypertension, anxiety, dizziness, constipation	Steatorrhea, oily spotting, faecal urgency	Nausea, diarrhoea, constipation, vomiting, headache, dyspepsia, cholelithiasis	Nausea, constipation, headache, vomiting, dizziness, insomnia, dry mouth, diarrhoea, hypertension	As for liraglutide
Mean placebo‐subtracted weight loss	7.4 kg over 36 weeks^14^	4% at 52 weeks^15^	4–6% at 56 weeks^16^	5% at 56 weeks^17^	12–14% at 68 weeks^18^, ^19^
Proportion of clinical trial participants with 5% and 10% weight loss at ~12 months	NA	73% and 41% (*v* 45% and 21% placebo)^20^	63% and 33% (*v* 27% and 11% placebo)^16^	48% and 25% (*v* 16% and 7% placebo)^17^	86% and 69% (*v* 32% and 12% placebo)^18^
Effects on reward‐related drivers of eating	Reduced craving for fats and sweets *v* placebo (Food Craving Inventory) at 12 weeks^21^	No difference in changes to eating restraint, disinhibition, or binge eating *v* placebo after 18–33 months (Three Factor Eating Questionnaire, Binge Eating Scale)^22^	Reduced desire to consume sweet, salty, fatty and savoury foods *v* placebo (visual analogue scales) at 16 weeks^23^	Reduced desire to consume sweet and starchy foods, reduced incidence and strength of food cravings, reduced eating in response to food cravings, increased ability to resist food cravings and control eating during 56 weeks (Control of Eating Questionnaire). No difference *v* placebo on Food Craving Inventory^24^	Improved control of eating, reduced incidence and strength of food cravings *v* placebo (Control of Eating Questionnaire) following 20 weeks of treatment^26^
				Altered activation *v* placebo in several brain areas in response to palatable food cues on functional MRI after four weeks of treatment^25^	
Effect on health‐related quality of life	NA	No difference in quality of life compared with lifestyle intervention plus placebo^27^	Greater improvement in all domains (IWQOL‐Lite) *v* placebo at 12 months^16^	Greater improvement in all domains (IWQOL‐Lite) *v* placebo at 12 months from week 8 of treatment^17^	Greater improvement in physical function (IWQOL‐Lite) *v* placebo and greater increase in mental component summary *v* placebo (SF‐36)^18^
Approximate cost in 2023 per month at maximum dose	$145	$93	$387	$240	NA
Other considerations	It is recommended that phentermine be used with caution, and with monitoring of blood pressure, in people with hypertension	Reduction in risk of developing type 2 diabetes by 37% *v* placebo in people at high risk at four years^20^	Reduction in risk of developing type 2 diabetes by 66% *v* placebo in people at high risk over three years^28^	No improvement in blood pressure with weight loss	
				Caution and reduced dosing in patients treated with antidepressants and some antipsychotics	

GLP‐1 = glucagon‐like peptide 1; IWQoL‐Lite = Impact of Weight on Quality of Life‐Lite; MAOI = monoamine oxidase inhibitors; MRI = magnetic resonance imaging; NA = data not available; POMC = pro‐opiomelanocortin; SF‐36 = 36‐Item Short Form Health Survey; SSRI = selective serotonin reuptake inhibitor.

* Several brands of phentermine, including Duromine, Metermine (iNova), and Phentermine Juno (Juno).

† Xenical (Pharmaco), Prolistat (Advanz Pharma).

‡ Saxenda (Novo Nordisk).

§ Contrave (iNova).

¶ Wegovy (Novo Nordisk).

### Orlistat

Orlistat reduces the absorption of dietary fats by preventing their digestion through the inactivation of gastric and pancreatic lipases, leading to an increase in excretion of up to 35% of ingested fat in the faeces.^29^ In clinical trials with at least 12 months’ follow‐up, the use of orlistat leads to weight loss of around 4–10 kg over 12 months (~3% in excess of placebo).^7^, ^30^

Gastrointestinal adverse effects such as steatorrhea (30% in clinical trials^15^), faecal urgency and oily leakage^31^ are common and related to the fat content of the meal. It is recommended that people taking orlistat also take a multivitamin supplement due to reduced absorption of fat‐soluble vitamins,^32^ although clinically significant vitamin deficiency is rarely reported.^30^ There are also reports of acute kidney injury in association with orlistat use due to hyperoxaluria and oxalate nephropathy.^33^ In Australia, orlistat is available from pharmacies without a prescription.

### Phentermine

Phentermine is a sympathomimetic agent. Studies in rats show that it stimulates the release of noradrenaline, dopamine and serotonin in several areas of the brain, including the hypothalamus.^34^, ^35^ Phentermine reduces hunger and reward‐related eating.^21^ It has been in use for more than 60 years (approved by the United States Food and Drug Administration [FDA] in 1959), and is the subject of few randomised controlled trials. Phentermine is indicated as a short term adjunct in the management of overweight and obesity (duration unspecified, often interpreted as < 12 weeks). A clinical trial of phentermine 30 mg daily reported weight loss of 12.2 kg (*v* 4.8 kg with placebo) in participants who completed 36 weeks of treatment.^14^

Common adverse effects of phentermine include insomnia, dry mouth, and increased blood pressure and heart rate. Cardiac valvular regurgitation was reported in people who took phentermine in combination with fenfluramine or dexfenfluramine (no longer available) for weight loss. Even though there are no reported cases to date of valvular heart disease associated with phentermine monotherapy, the combination of phentermine with selective serotonin reuptake inhibitors is not recommended due to the theoretical risk of cardiac valvular disease.^36^

### Naltrexone–bupropion

The combination of the antidepressant bupropion and the opioid antagonist naltrexone was developed for obesity management based on the synergistic effects of these agents in central appetite and reward regions. Bupropion is a dopamine and noradrenaline reuptake inhibitor that stimulates the activity of anorexigenic pro‐opiomelanocortin (POMC) neurons in the hypothalamus.^24^ Naltrexone blocks endogenous opioid‐mediated inhibition of POMC neurons, leading to sustained POMC stimulation.^24^ Clinical trials report weight loss of 5–6 kg (3–5% above placebo) over one year.^17^, ^37^, ^38^

The adverse effects most commonly reported in clinical trials of naltrexone–bupropion are nausea (30%) and constipation (16%), particularly during dose escalation.^37^ Increased blood pressure and heart rate are also potential adverse effects. The label contains a warning about an increased risk of depression and suicidal behaviour based on these effects with bupropion monotherapy, although clinical trials have not reported increased depression or suicidality with the naltrexone–bupropion combination compared with placebo.^17^, ^37^, ^38^

Clinicians should be aware of several potential drug interactions when prescribing naltrexone–bupropion. Concomitant treatment with certain cytochrome P450 2B6 (CYP2B6) inhibiting agents (eg, clopidogrel) can increase bupropion exposure. Conversely, CYP2B6 inducers (including human immunodeficiency virus antivirals and some anticonvulsant medications, such as carbamazepine and phenytoin) may reduce efficacy by increasing bupropion metabolism. Bupropion inhibits cytochrome P450 2D6 (CYP2D6) thereby reducing metabolism of some antidepressant (selective serotonin reuptake inhibitors and tricyclics), antipsychotic (eg, haloperidol, risperidone) and cardiac medications (eg, metoprolol, flecainide). Extreme caution is advised when combining naltrexone–bupropion with drugs that lower the seizure threshold (including some antipsychotic and antidepressant agents). In addition, central nervous system toxicity has been reported with concomitant use of bupropion with dopaminergic drugs such as levodopa.^39^

### Glucagon‐like peptide 1 receptor agonists

GLP‐1 is a hormone secreted by L cells in the distal small intestine and colon in response to ingestion of nutrients. Its numerous metabolic actions are mediated via GLP‐1 receptors, which are widely distributed, including in the brain, pancreas, stomach, kidney, heart, and adipose tissue.^40^ GLP‐1 receptor agonists reduce food intake by acting at GLP‐1 receptors in appetite‐ and reward‐related regions of the brain, including the hypothalamus, hindbrain and mesolimbic pathway, to promote satiation and reduce hunger and food reward.^40^ Additional effects of GLP‐1 include stimulation of insulin secretion under conditions of hyperglycaemia, reduction in glucagon secretion, and slowing of gastric emptying.^41^, ^42^

Endogenous GLP‐1 has a short half‐life of one to two minutes, as it is rapidly inactivated by the enzyme dipeptidylpeptidase‐4 (DPP‐4) and cleared from the circulation by the kidneys. GLP‐1 receptor agonists have been developed to promote the favourable metabolic effects of native GLP‐1 with longer duration of action and greater bioavailability.

Several GLP‐1 receptor agonists are available in Australia for the treatment of type 2 diabetes, of which two, liraglutide and semaglutide, are also indicated for obesity treatment at higher doses (liraglutide 3.0 mg daily and semaglutide 2.4 mg weekly for obesity, and up to 1.8 mg daily and 1.0 mg weekly, respectively, for type 2 diabetes). When used at recommended doses for obesity management in people without type 2 diabetes, mean weight losses in clinical trials are 6–8 kg (~6% placebo‐subtracted) for liraglutide^16^ and 15–18 kg (~13% placebo‐subtracted) with semaglutide.^18^, ^19^

The adverse effects of GLP‐1 receptor agonists are predominantly gastrointestinal. Nausea (40%) and diarrhoea (21%) are the most common adverse effects for liraglutide 3.0 mg,^16^ with a similar adverse event profile for semaglutide 2.4 mg.^43^ A gradual dose escalation is recommended to minimise these effects. Nausea is usually transient and mild and is most common shortly after treatment initiation and during dose escalation.^16^, ^18^

In people with type 2 diabetes, the 1 mg dose of semaglutide for type 2 diabetes treatment was associated with a higher risk of complications of diabetic retinopathy in patients with a history of retinopathy at the time of treatment initiation.^44^ This might be related to rapid improvement in glycaemic control, which is known to be associated with transient worsening of diabetic retinopathy.^45^ Hence, retinopathy‐related risks are likely to be much lower in people without diabetes. The effect of semaglutide (up to 1 mg weekly) on the development and progression of diabetic retinopathy over five years will be examined in a dedicated trial (ClinicalTrials.gov, NCT03811561). Some epidemiological studies have suggested an increased risk of acute pancreatitis and pancreatic cancer in people using GLP‐1 receptor agonists. However, a meta‐analysis of more than 55 000 participants from cardiovascular outcome trials in people with type 2 diabetes using GLP‐1 receptor agonists (including semaglutide and liraglutide) at doses used to treat type 2 diabetes did not detect a signal for these events over 175 000 patient‐years of observation.^46^

### Topiramate

Topiramate is a carbonic anhydrase inhibitor indicated for the treatment of epilepsy and as prophylaxis against migraines. When used in the treatment of epilepsy, anorexia and weight loss are common adverse effects. The mechanism by which topiramate decreases appetite is not known.

In Australia, topiramate is not approved by the TGA for an obesity indication, but it is inexpensive and commonly used “off‐label” for obesity management, either as monotherapy or in combination with medications, particularly phentermine.^47^ The combination of phentermine with an extended‐release formulation of topiramate is approved for chronic weight management in the United States but not in Australia. Of note, the recommended dose of this combination contains 7.5 mg phentermine and 46 mg of topiramate (half of the minimum available capsule size of phentermine in Australia), with a maximum dose of 15 mg phentermine plus 92 mg topiramate.

Gradual dose escalation of topiramate is recommended to improve tolerability, starting with 12.5 mg once a day and increasing to a maximum of 50 mg twice a day. Common adverse effects include paraesthesia, dysgeusia (taste distortion), somnolence, memory, attention and concentration difficulties, and mood disturbances.^48^ Rare adverse effects include kidney stones and angle closure glaucoma. Topiramate is contraindicated in pregnancy due to an association with congenital malformations. A meta‐analysis of randomised trials examining the effect of topiramate use for 16 weeks or more on weight loss reported a mean placebo‐subtracted weight loss of 5.3 kg.^48^

### Variability in weight loss responses

Medications indicated for obesity management are consistently associated with lower weight losses in people with type 2 diabetes than in those without diabetes.^16^, ^18^, ^49^, ^50^ The reasons for this are not known but might include concomitant use of glucose‐lowering agents that promote weight gain, increased food intake to prevent or treat hypoglycaemia, and reduction in glycosuria (due to glycaemic improvement) offsetting weight loss.^51^

Although mean weight loss with current obesity medications (with the exception of semaglutide) is in the range of 3–6% in excess of placebo, it is important to note that individual responses vary widely for all agents, with 25–69% of participants achieving at least 10% weight loss over one year of treatment.

### Health improvements associated with obesity medications

All obesity medications are associated with improvements in cardiovascular risk factors, although these benefits differ between medications even if similar mean weight loss is achieved. For example, low‐density lipoprotein cholesterol lowering is greater with orlistat than other agents, which is likely due to its effect on reducing fat absorption.^20^, ^52^, ^53^ Weight loss induced by naltrexone–bupropion is not associated with the expected improvement in blood pressure.^24^, ^37^, ^38^ As anticipated from their pancreatic actions, GLP‐1 receptor agonists are associated with greater glycaemic improvements than other agents after a similar amount of weight loss.^17^, ^50^ Orlistat^54^ and GLP‐1 receptor agonists^55^, ^56^ appear to reduce liver fat content and markers of liver injury in people with non‐alcoholic fatty liver disease, but whether this effect is independent of weight loss remains unclear.^54^, ^57^ There is a lack of data on the effects of other obesity medications on non‐alcoholic fatty liver disease.^54^

As yet, there are no completed cardiovascular outcome trials of medications currently indicated for obesity management. Such trials have not been conducted for phentermine and orlistat, and a cardiovascular outcome trial of naltrexone–bupropion was terminated prematurely due to a breach of confidentiality regarding interim analyses.^58^ In people with type 2 diabetes, who have a higher cardiovascular risk than the general population, liraglutide and semaglutide have demonstrated reductions in major adverse cardiovascular outcomes compared with placebo (at doses used for the treatment of type 2 diabetes: liraglutide 1.8 mg daily; semaglutide 0.5 mg and 1.0 mg weekly).^44^, ^59^ In people without type 2 diabetes, an analysis of pooled data from five trials found that liraglutide 3.0 mg did not reduce cardiovascular events compared with placebo (*n* = 5064; hazard ratio, 0.48; 95% CI, 0.16–1.44).^60^ A cardiovascular outcome trial of semaglutide 2.4 mg weekly in 17 500 people is underway in people with obesity (but without type 2 diabetes) who have established cardiovascular disease.^61^

Most approved obesity medications have been shown to improve health‐related or weight‐related quality of life. Orlistat might not improve quality of life compared with placebo,^27^ and quality of life outcomes have not been reported for phentermine. All obesity medications except orlistat are associated with improvements in eating behaviour compared with placebo (Box 2).^21^, ^22^, ^23^, ^25^, ^26^

## Novel and emerging medications for obesity

### Tirzepatide

Tirzepatide is a dual agonist of receptors for both GLP‐1 and glucose‐dependent insulinotropic polypeptide (GIP). Following nutrient ingestion, these two gut hormones stimulate insulin secretion via GLP‐1 and GIP receptors on pancreatic β‐cells, thereby minimising postprandial blood glucose elevation. Receptors for both hormones are also found in regions of the brain that regulate food intake, although studies are mixed in their findings on the contribution of GIP receptor activation to food intake and body weight.^62^ Tirzepatide potently reduces body weight as well as blood glucose. It is approved by the FDA and the TGA for the treatment of type 2 diabetes at a once‐weekly subcutaneous dose of 5 mg, 10 mg and 15 mg and is undergoing clinical trials for the management of obesity at the same doses, as well as other conditions, such as heart failure with preserved ejection fraction and non‐alcoholic steatohepatitis.

Results from a phase 3 clinical trial for obesity (*n* = 2539) showed that tirzepatide 5 mg, 10 mg and 15 mg resulted in mean placebo‐subtracted weight loss over 72 weeks of 11.9%, 16.4% and 17.8% respectively (total weight loss of 15–20 kg).^63^ More than half of participants in the 10 mg and 15 mg groups lost 20% or more of body weight, and improvements were observed in blood pressure, lipid profile, glycaemia and physical function. The adverse effect profile appears similar to GLP‐1 receptor agonists, with predominantly gastrointestinal effects noted.^63^ At the time of writing, tirzepatide is indicated for type 2 diabetes but is not yet available in Australia.

### Cagrilintide

Amylin is a hormone co‐secreted with insulin from pancreatic β‐cells in response to nutrient ingestion. It slows the absorption of glucose into the circulation by delaying gastric emptying and acts at receptors in the hindbrain and mesolimbic dopamine system to increase satiation and reduce the rewarding value of food.^64^

Cagrilintide is a long‐acting amylin analogue in development for the management of obesity as a once weekly subcutaneous injection. Results from a phase 2 study indicate weight loss of up to 11 kg (8% greater than placebo and almost 2% greater than liraglutide 3.0 mg daily) over 26 weeks.^65^ A combination of cagrilintide and semaglutide is also being investigated for obesity management.^66^

### Setmelanotide

Setmelanotide is a melanocortin 4 receptor agonist developed for the treatment of rare monogenic obesity syndromes caused by defects in the hypothalamic leptin–melanocortin pathway. These disorders are characterised by severe hyperphagia and early‐onset obesity.^67^ Setmelanotide is approved for use in the US and Europe, but not currently Australia, for patients aged six years or older with POMC, proprotein convertase 1 or leptin receptor deficiency. Setmelanotide is also undergoing trials for use in other genetic disorders associated with obesity, such as Bardet–Biedl syndrome.^68^ Injection site reactions and skin pigmentation are the most common adverse effects noted in clinical trials.

### Bimagrumab

Bimagrumab is a human monoclonal antibody that binds to the activin type II receptor (ActRII). ActRII blockade prevents the binding of natural ligands that negatively regulate skeletal muscle growth and, in animal models, promotes brown adipose tissue differentiation and activity.^69^, ^70^ In a small 48‐week phase 2 trial in adults with type 2 diabetes and obesity, bimagrumab (10 mg/kg up to 1200 mg, intravenous infusion every four weeks) resulted in loss of fat mass of 20.5% compared with 0.5% in the placebo group (7.5 kg *v* 0.2 kg respectively), and a gain in lean mass of 3.6% compared with loss of 0.8% in the placebo group (+1.7 kg *v* ‐0.4 kg respectively) as well as improved glycaemic control.^71^ The increase in lean mass is notable, as negative energy balance and weight loss are typically associated with lean mass reduction. The mechanism for fat mass reduction is unknown. Diarrhoea and muscle spasms were the most common adverse effects.

### Other gut hormone‐based agents

Several other medications with dual and triple action at gut hormone receptors (eg, GLP‐1, GIP, glucagon, amylin) are under development.^72^, ^73^ These agents aim to exploit the complementary actions of these hormones on appetite and glycaemia and mimic the endogenous release of several gut hormones postprandially.

## Considerations for use of obesity medications in clinical practice

### Concurrent lifestyle intervention

The main purpose of obesity treatment is to improve health and wellbeing. All medications are indicated in conjunction with lifestyle interventions, as optimising diet quality, reducing energy intake and increasing energy expenditure have their own health benefits, as well as improving the effectiveness of treatment with obesity medications.^74^, ^75^ As an example, the use of liraglutide in combination with physical activity over 12 months resulted in an additional mean weight loss of 2.7 kg and reduction in body fat of 1.7% compared with liraglutide alone.^75^

### Timing and duration of treatment

Use of medications should be considered at the initiation of an obesity management program, particularly if there have been previous unsuccessful attempts at lifestyle interventions alone. Where the primary treatment modality is lifestyle interventions or bariatric surgery, the addition of medications might be useful if therapeutic goals have not been reached, or to prevent or reduce weight regain.^76^

When obesity medications are initiated, and while they are in use, blood pressure should be monitored and doses of antihypertensive agents adjusted as required. In people with type 2 diabetes, glucose‐lowering medications might need to be reduced to avoid hypoglycaemia, particularly if a GLP‐1 receptor agonist is added.

Current TGA recommendations state that obesity medications should be discontinued if loss of at least 5% of total body weight has not occurred after 12 weeks of use at the maximal dose. Since early response to treatment is associated with long term weight outcomes, this recommendation is aimed at preventing prolonged use of an ineffective treatment for weight reduction. However, it does not take into account the benefit of preventing recurrence of obesity‐related complications if a medication is initiated for prevention or mitigation of weight regain.

As is the case for medications used to treat most chronic diseases, medications for obesity are only effective while in use.^43^, ^77^ Therefore, long term use is likely to be required for sustained benefits to health and health‐related quality of life, although data on long term safety and clinical outcomes for obesity medications are currently limited.^78^, ^79^ Adherence to treatment is a challenge. One‐year treatment discontinuation rates in clinical trials are 17–50% for medications currently approved for long term weight management,^15^, ^16^, ^18^, ^37^ but persistence with treatment is much lower (< 10%) in real‐world studies.^79^

### Choice of agent

To date, few studies have directly compared outcomes of different obesity medications. Randomised trials have shown greater mean weight loss with liraglutide 3 mg daily compared with orlistat 120 mg three times a day (mean difference, 3.7 kg at one year)^80^ and with semaglutide 2.4 mg weekly compared with liraglutide 3.0 mg daily (mean difference, 8.5 kg at week 68).^19^

There is a suggestion that patients with certain biological and behavioural characteristics might respond better to particular medications,^81^ but data are currently insufficient to guide clinical practice. Choice of agent is currently based largely on the expected benefits and adverse effect profiles of each agent in relation to the individual patient as well as patient preferences (including for mode of administration), medication availability and cost.

As with other chronic diseases, the use of more than one agent with complementary mechanisms of action might be more effective, and associated with fewer side effects, than monotherapy. This approach has not been studied in clinical trials combining the currently available agents but is being explored for new medications in development.^66^

### Access to treatment

Only a minority of eligible people (estimated at 1.3% in a cohort of > 2 million US adults) are treated with obesity medications.^82^ There are a number of likely contributing factors, including out‐of‐pocket costs, lack of recognition of the role of medications in the management of obesity, a perception that mean weight losses associated with most agents are insufficient to justify their use, a lack of long term clinical trial data, and safety concerns following the withdrawal from the market of several older classes of medications.^82^ Stigma associated with obesity and its treatment is another potential contributor, as many of these concerns are less often raised for medications, even new agents, for the treatment of other chronic diseases.^83^

## Conclusion

Five medications are currently indicated for obesity management in Australia, all of which have beneficial effects on obesity‐related complications. The newest agents, and those in clinical development, are associated with considerably greater mean efficacy than older agents, but interindividual variability in response to all agents is substantial. When medications are used, important considerations include treatment goals, patient preferences, weight‐loss‐independent benefits of individual agents, contraindications, side effect profiles, method of administration, and costs. The effective management of obesity requires a long term approach.

## Open access

Open access publishing facilitated by The University of Melbourne, as part of the Wiley ‐ The University of Melbourne agreement via the Council of Australian University Librarians.

## Competing interests

Priya Sumithran has co‐authored manuscripts which have had medical writing assistance provided (Novo Nordisk). No medical writing assistance was received for this manuscript. Priya Sumithran is in the leadership group of the Obesity Collective.

## Provenance

Commissioned; externally peer reviewed.
